# Prediction of Metastasis in the Axillary Lymph Nodes of Patients With Breast Cancer: A Radiomics Method Based on Contrast-Enhanced Computed Tomography

**DOI:** 10.3389/fonc.2021.726240

**Published:** 2021-09-20

**Authors:** Chunmei Yang, Jing Dong, Ziyi Liu, Qingxi Guo, Yue Nie, Deqing Huang, Na Qin, Jian Shu

**Affiliations:** ^1^Department of Radiology, The Affiliated Hospital of Southwest Medical University, Luzhou, China; ^2^Nuclear Medicine and Molecular Imaging Key Laboratory of Sichuan Province, The Affiliated Hospital of Southwest Medical University, Luzhou, China; ^3^The Institute of Systems Science and Technology, Southwest Jiaotong University, Chengdu, China; ^4^Department of Pathology, The Affiliated Hospital of Southwest Medical University, Luzhou, China; ^5^Department of Radiology, Luzhou People’s Hospital, Luzhou, China

**Keywords:** breast cancer, metastasis, axillary lymph node, radiomics, computed tomography

## Abstract

**Background:**

The use of traditional techniques to evaluate breast cancer is restricted by the subjective nature of assessment, variation across radiologists, and limited data. Radiomics may predict axillary lymph node metastasis (ALNM) of breast cancer more accurately.

**Purpose:**

The aim was to evaluate the diagnostic performance of a radiomics model based on ALNs themselves that used contrast-enhanced computed tomography (CECT) to detect ALNM of breast cancer.

**Methods:**

We retrospectively enrolled 402 patients with breast cancer confirmed by pathology from January 2016 to October 2019. Three hundred and ninety-six features were extracted for all patients from axial CECT images of 825 ALNs using Artificial Intelligent Kit software (GE Medical Systems, Version V3.1.0.R). Next, the radiomics model was trained, validated, and tested for predicting ALNM in breast cancer by using a support vector machine algorithm. Finally, the performance of the radiomics model was evaluated in terms of its classification accuracy and the value of the area under the curve (AUC).

**Results:**

The radiomics model yielded the best classification accuracy of 89.1% and the highest AUC of 0.92 (95% CI: 0.91-0.93, *p*=0.002) for discriminating ALNM in breast cancer in the validation cohorts. In the testing cohorts, the model also demonstrated better performance, with an accuracy of 88.5% and an AUC of 0.94 (95% CI: 0.93-0.95, *p*=0.005) for predicting ALNM in breast cancer.

**Conclusion:**

The radiomics model based on CECT images can be used to predict ALNM in breast cancer and has significant potential in clinical noninvasive diagnosis and in the prediction of breast cancer metastasis.

## Introduction

Breast cancer is the most frequently diagnosed cancer in females and accounts for 29% of all new cancer diagnoses in women, with an estimated 1.7 million cases annually (1, 2). General trends indicate that the incidence and mortality rates of breast cancer have increased gradually during the past decade (3). Axillary lymph node metastasis (ALNM) is the most important lymphatic metastatic pathway for breast cancer. The status of the axillary lymph nodes (ALNs) is associated with a poor prognosis (4) and is highly significant in judging the stage of breast cancer and selecting the optimal treatment strategy, in particular the adjuvant treatment plan after surgery (5). Therefore, it is critical to evaluate ALNM of breast cancer in a noninvasive and accurate way, as it may guide optimal treatment decisions and determine the prognosis.

At present, the gold standard of diagnosis for ALNM of breast cancer remains pathological examination. ALNM may be confirmed by ultrasound-guided fine needle aspiration, axillary lymph node dissection (ALND), or sentinel lymph node dissection (SLND). Among these methods, SLND has become an alternative to ALND and is recommended for dealing with nonpalpable ALN (6). However, these are invasive methods, with potential complications of arm pain, hematoma, seroma, lymphedema, and infection (6). Traditional imaging methods, including ultrasonography (US) (7), mammography (8), computed tomography (CT) (9), positron emission tomography/computed tomography (PET/CT) (10), and magnetic resonance imaging (MRI) (11), have all been used as noninvasive methods for evaluating the status of ALN in patients with breast cancer. However, some drawbacks to their use exist. Conventional techniques rely on a subjective visual evaluation by the radiologist and have a lower diagnostic sensitivity, which may lead to a considerable proportion of false positive and negative rates (12). Therefore, there is currently still a need for quantitative and noninvasive methods that can predict ALNM of breast cancer.

Radiomics, a recently introduced methodology, extracts quantitative features from traditional images, including X-ray, CT, MRI, PET/CT, or US images, and has been used to develop a radiomics model to quantify the heterogeneity of lesions objectively, especially in oncology patients (13). In several recent studies radiomics has been successfully employed to preoperatively evaluate lymph node metastasis (LNM) in some types of cancers (14, 15). Radiomics models have also been used to predict LNM of breast cancer and determine its prognostic value (16–24). However, all of these studies were based on the texture features of tumor tissues to identify whether local lymph nodes were metastatic. To our knowledge, there has been no publication that has determined whether the radiomics features of ALNs would render a better prediction of ALNM in patients with breast cancer.

The aim of our study was to develop a radiomics model for ALNs by using a support vector machine (SVM) algorithm based on contrast-enhanced CT (CECT) images to quantitatively predict ALNM of breast cancer. Fortunately, our model demonstrated promising prediction performance and may facilitate clinical decision making and improve survival outcomes in selected patients.

## Materials and Methods

### Patient Characteristics

In our study, all procedures involving human subjects were conducted in accord with the Declaration of Helsinki, and ethical approval (KY2019182) for this study was acquired from the Ethical Committee of our hospital. The informed consent of patients was waived because the study was retrospective and completely anonymized the data of all patients.

The inclusion criteria were: (1) patients had presurgical CECT images available and (2) patients underwent surgical excisions (including tumor resection and ALND) and pathological examinations. The exclusion criteria were: (1) existence of some small ALNs (short diameter of ALNs < 0.50 cm) with the region of interest (ROI) being delineated with difficulty in CECT images and (2) existence of some ALNs whose pathological results failed to match their positions in the CECT images. Ultimately, a total of 402 patients with a pathological diagnosis of breast cancer were retrospectively included in our study from January 2016 to October 2019.

### Clinical and Pathological Information

The following clinical characteristics and CT manifestations of patients, including age, disease stage, type of surgery, tumor grading, long diameter of the ALN, short diameter (measured on the section showing the maximum size of the ALN in axial CECT images), and the status of the fatty hilum in the ALNs, were collected and recorded. Meanwhile, pathological and immunohistochemical characteristics, including the status of ALNs (metastasis or non-metastasis), the number and order of size of metastatic ALNs, estrogen receptor (ER) status, progesterone receptor (PR) status, human epidermal growth factor receptor 2 (HER-2) status, Ki-67 levels, and histological tumor type, were obtained. Tumors were considered ER or PR positive if ≥ 10% of the cells stained positive and HER-2 positivity was defined as hematoxylin-eosin staining of at least 3 + (19). Ki-67 positivity was defined as a proliferation index ≥ 14%, and otherwise was considered negative (19).

**Figure 1:**
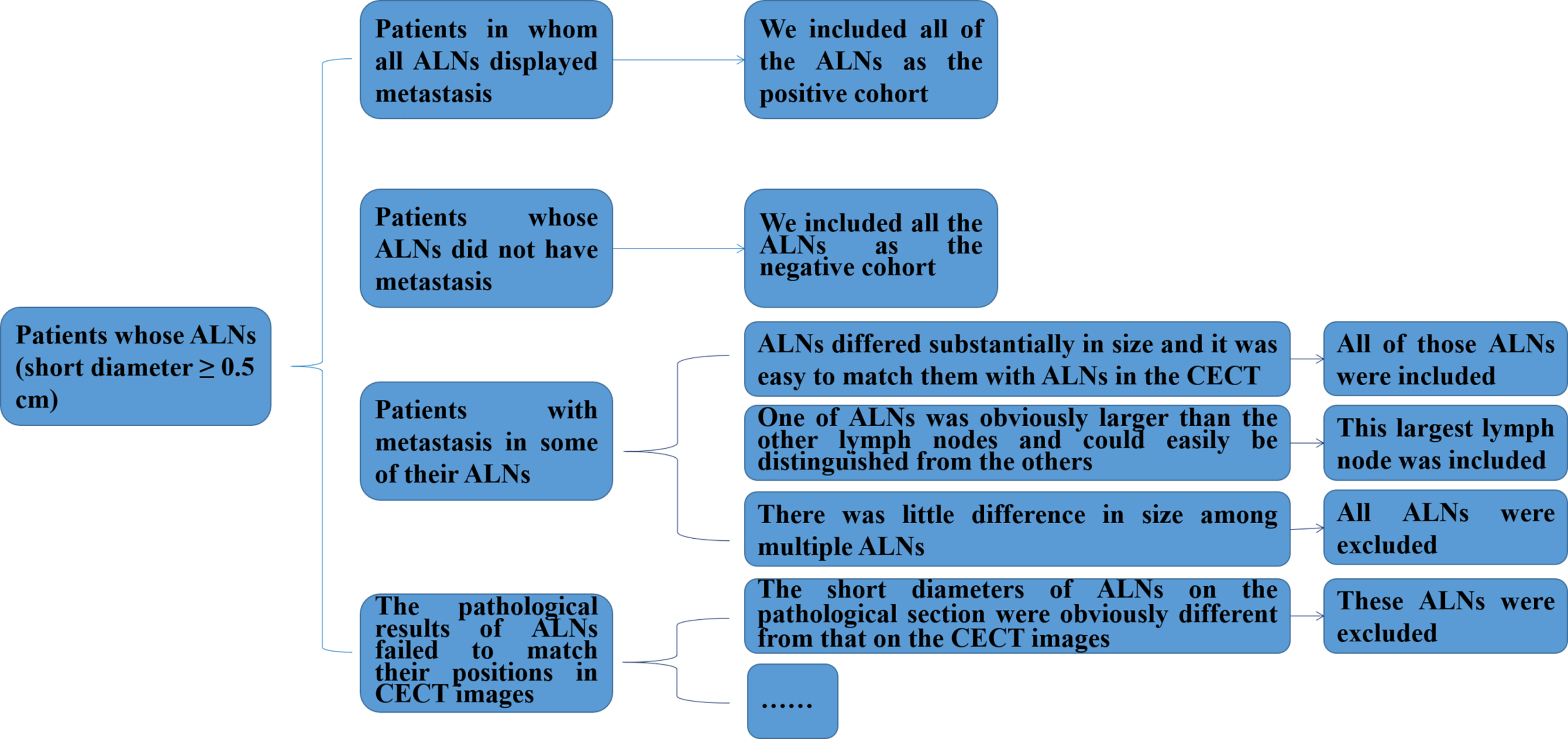
Flowchart of the inclusion and exclusion criteria for ALNs in patients with breast cancer in this study. ALNs, axillary lymph nodes; CECT, contrast-enhanced computed tomography.

In particular, we not only recorded the positions of ALNs during the surgery, but also ranked ALNs in a descending order according to the size of the ALN in the pathological sections and labeled them 1, 2, 3 etc. for patients with pathological diagnosis of ALNM. Then, for each patient, two pathological experts with 10 and 3 years of experience each reviewed the corresponding status (metastasis or non-metastasis) of all ALNs with a short diameter of ≥ 0.5 cm and recorded them in order according to the same procedure. **Figure 1** shows a flowchart of the inclusion and exclusion criteria for ALNs in patients with breast cancer in our study. More details are described in the **Supplementary Material**.

### CECT Acquisition Protocol

All patients recruited in this study underwent preoperative thoracic CECT scanning. CECT images were obtained from different CT scanners, including a Brilliance iCT 256 (Philips Healthcare, Cleveland, OH) and GE LightSpeed 64-slice CT (GE Medical systems, Milwaukee, WI). The scanning method was as follows: the contrast agent (Iohexol, 350 mg/mL) was injected using a high pressure syringe through the median cubital vein of the patients (the dosage was 2.0 mL/kg, and the flow rate was 3.0 mL/s). The CT value in the blood vessel at the level of the aortic arch was monitored after the injection of the contrast agent. Enhanced CT scanning started when the CT value reached about 250 HU. The scanning range extended from the level of the lower neck to the bottom of the thorax in the supine position. The detailed parameters and scheme are shown in the **Table 1**.

**Table 1:** Parameters of thoracic CECT scanning.

	Philips Brilliance iCT 256	GE LightSpeed 64-slice CT
Matrix	512 x 512	512 x 512
Slice thickness (mm)	5	5
Pitch (mm)	1	1
Tube voltage (kV)	120	120
Tube current (mA)	250	400

### CECT Imaging Process

All CECT images for patients were retrieved from the Picture Archiving and Communication System and saved as Digital Imaging and Communications in the Medicine format. Given that these images were obtained from different CT scanners, we first used Artificial Intelligent Kit (AK) software (GE Medical systems, Version V3.1.0.R) to conduct image preprocessing. Resampling, Gaussian, and normalization algorithms were adopted to normalize the image intensities, which minimized the influence of contrast and brightness variations.

### Image Segmentation

**Figure 2:**
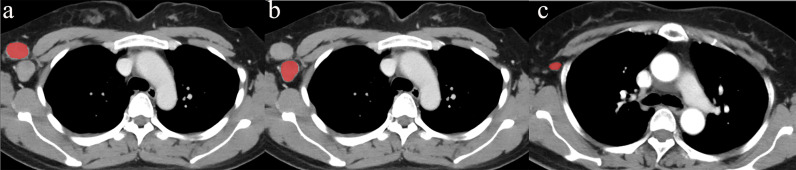
Delineation of the ROI. The ROI was located on the maximum section of the ALN, avoiding the fatty hilum of the lymph node on the axial CECT images. To minimize volume averaging with adjacent structures, the line was drawn carefully to maintain an approximate distance of 1–2 mm from the lymph node margin on the CECT images. **(A)** and **(B)** showed the delineation of two metastatic ALNs. **(C)** showed the delineation of a non-metastatic ALN.

First, for all patients with short diameters of ALNs ≥ 0.5 cm, a radiologist with 8 years of experience and a pathologist with 10 years of experience matched the pathological result of each ALN with its position in the CECT image. ROI segmentation was performed on the section showing the maximum size of the ALN in the axial CECT images (window width was 350 HU and window level was 35 HU) for all patients, avoiding the fatty hilum of the ALN. The radiologist, who was blinded to the patients’ characteristics, delineated the ROIs of the ALNs manually (**Figure 2**) using freely available software (ITK-SNAP, http://www.itksnap.org). To minimize volume averaging with adjacent structures, a line was drawn carefully to maintain an approximate distance of 1-2 mm from the lymph node margins in the CECT images.

### Extraction of Radiomics Features

Radiomics features were extracted from the axial CECT images which were anonymous using AK software. A total of 396 quantitative radiomics features were automatically extracted from each ROI. These features were categorized into six groups, including histogram, gray-level co-occurrence matrix, run length matrix, gray level size zone matrix, formfactor, and haralick features.

### Intraobserver and Interobserver Agreement

To evaluate the reproducibility of the radiomics features, we randomly chose 100 ALNs delineated from the axial CECT images by two experienced radiologists (radiologist 1 and radiologist 2, with 8 and 5 years of chest CT diagnosis experience, respectively). For the intraobserver agreement, radiologist 1 delineated an ROI twice within 2 weeks following the same steps. Meanwhile, radiologist 2 also independently drew the ROI once according to the same steps and then the interobserver reproducibility was evaluated by comparing the results with the features obtained from the first ROI delineation by radiologist 1. Inter- and intraclass correlation coefficients (ICC) were calculated, and generally, an ICC > 0.75 indicated high reproducibility.

### Training, Validation, and Testing of the Radiomics Model

First, all radiomics features were normalized to eliminate the adverse effects caused by the singular sample data using the Min-Max Normalization method [V_i_ = (V_i_ - Min)/(Max - Min)], where V_i_, Min, and Max are the feature, the minimum feature, and the maximum feature, respectively. Then, we randomly selected 20% of the positive samples (ALNs with metastasis, n = 80) and 20% of the negative samples (ALNs without metastasis, n = 85) as the testing set (n = 165).

The remaining 80% of the positive samples (n = 320) and 80% of the negative samples (n = 340) were randomly mixed and divided into the training set (n = 495, including 240 positive samples and 255 negative samples) and validation set (n = 165, including 80 positive samples and 85 negative samples) based on a ratio of 3:1. This step was randomly repeated 8,000 times.

Finally, the training set, validation set, and testing set were obtained according to the ratio of samples of 3:1:1.

**Figure 3:**
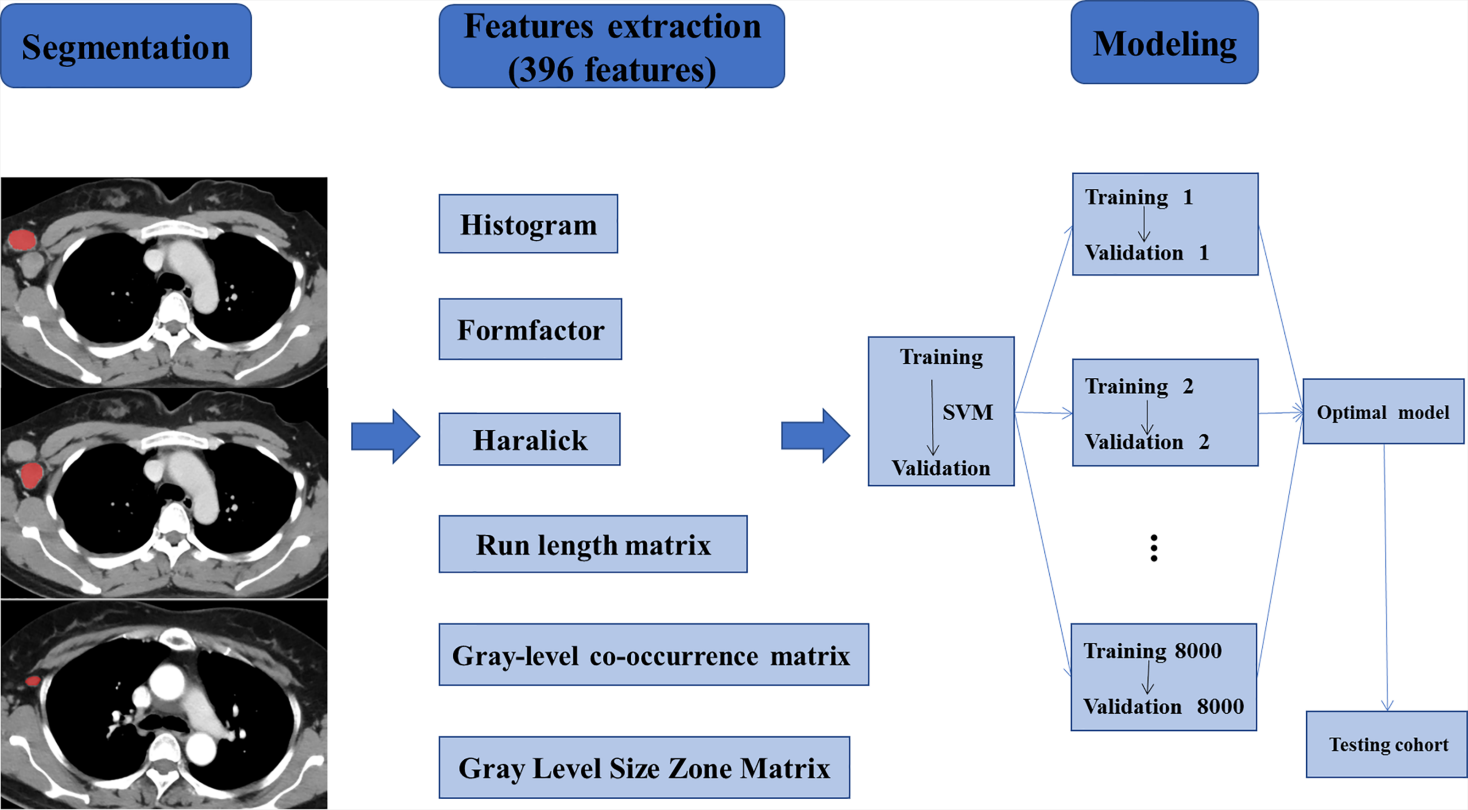
Workflow of the necessary steps in the study. ALNs were segmented manually on axial CECT images. 396 radiomics features, divided into six groups, were extracted from within the defined ALN contours on CECT images to quantify the texture of the lymph nodes. The radiomics model was trained and validated based on the support vector machine (SVM) algorithm. Training and validation were repeated 8000 times to ensure good reliability and achieve the optimal prediction model. Finally, a new testing set was used to verify the established model.

Next, the radiomics model was constructed based on the support vector machine (SVM, linear kernel function, default settings) algorithm for predicting ALNM after comparison with other kernels, e.g. the polynomial kernel function and radial basis kernel function. We randomly selected the training cohorts and the validation cohorts each time, and the training set was used to train the radiomics model and the validation set was used to verify the classification accuracy of the model. We repeated this step 8,000 times to ensure good reliability. Afterward, average and maximum classification accuracies in the validation cohorts were determined. Next, we acquired the radiomics model based on SVM with the maximum classification accuracy in the validation cohorts for further verification. Finally, a new testing set was used to test the radiomics model with the maximum classification accuracy in the validation cohorts. The flowchart of the study is shown in **Figure 3**.

### Statistical Analysis

Continuous data (age, long and short diameters of ALNs) were expressed as the mean ± standard deviation (SD) when the distribution of data was normal, or as the median (interquartile range) when they disobeyed normality. Given the distribution of these data, the above variables were compared using independent *t-*tests or Wilcoxon rank sum tests, as appropriate. Categorical variables, including the shape, the status of the fatty hilum, ER status, PR status, HER-2 status, and Ki-67 levels, were compared using cross-tabulation. The above statistical analyses were performed using SPSS 17.0. The discrimination metrics of the established radiomics model, including the area under the curve (AUC) for the receiver operating characteristic (ROC), classification accuracy, sensitivity, specificity, positive predictive value (PPV), and negative predictive value (NPV), were calculated using Python (Version 3.7.2, https://scikit-learn.org/dev/index.html). A two-sided *p* value < 0.05 was considered statistically significant.

## Results

### Patient Characteristics

The baseline characteristics of all patients are included in **Table 2**. A total of 402 patients with breast cancer were enrolled in our study with a mean age of 51.5 ± 8.9 years, ranging from 26 to 81 years. A total of 188 patients were diagnosed with ALNM, and 90 of those had 1-2 metastatic ALNs and 98 had more than two metastatic ALNs. The remaining 214 patients were diagnosed with no ALNM, among which 83 had only one ALN and the remaining 131 had two or more ALNs to be included in our study. We discovered that the Ki-67 level (*p* = 0.009) were significantly different between patients with ALNM and the non-ALNM group. However, patient age, ER status, PR status, and HER-2 status were not significantly different between the positive and negative groups.

**Table 2:** Clinical, pathological, and immunohistochemical characteristics in patients with breast cancer.

Characteristics	The patients with ALNs status (n = 402)		
	Positive (n = 188)	Negative (n = 214)	*p* value
Age (mean ± SD, years)	51.0 ± 9.0	52.1 ± 8.9	0.219
ER status (%)			0.466
Positive	126 (31.3)	136 (33.8)	
Negative	62 (15.4)	78 (19.5)	
PR status (%)			0.855
Positive	108 (26.9)	121 (30.1)	
Negative	80 (19.9)	93 (23.1)	
HER-2 status (%)			0.258
Positive	68 (16.9)	66 (16.4)	
Negative	120 (29.9)	148 (36.8)	
Ki-67 level (%)			0.009
Positive	156 (38.8)	154 (38.3)	
Negative	32 (8.0)	60 (14.9)	

ALNs, axillary lymph nodes; ER, estrogen receptor; HER-2, human epidermal growth factor receptor 2; PR, progesterone receptor; SD, standard deviation. P < 0.05 was considered statistically significant.

Eventually, a total of 825 ALNs were included in our study. Based on the pathological examination of the ALNs, 400 were confirmed as metastasis positive. The other 425 ALNs were confirmed as negative. Details of the ALN characteristics are presented in **Table 3**. Among the characteristics of all ALNs, the long diameter (*p* < 0.001), short diameter (*p* < 0.001), status of the fatty hilum (*p* < 0.001), and shape (*p* < 0.001) were significantly correlated with the ALN status.

**Table 3:** Clinical and imaging manifestations of ALNs in patients with breast cancer.

Characteristics	ALNs status (n = 825)		
	Positive (n = 400)	Negative (n = 425)	*p* value
Long diameter (cm)	1.34 (1.03, 1.68)	1.01 (0.83, 1.30)	0.000
Short diameter (cm)	0.94 (0.75, 1.24)	0.69 (0.58, 0.88)	0.000
Shape (%)			0.000
Round/Oval	376 (45.6)	325 (39.4)	
Irregular	24 (2.9)	100 (12.1)	
The status of fatty hilum (%)			0.000
Positive	42 (5.1)	257 (40.2)	
Negative	358 (43.4)	168 (11.3)	

ALNs, axillary lymph nodes. Long diameter and short diameter of ALNs were expressed as medians (upper and lower quartiles) because the distribution of data was outside the bounds of normality. P < 0.05 was considered statistically significant.

### Intraobserver and Interobserver Agreement

Upon determining the reproducibility of feature extraction, 396 radiomics features achieved favorable consistency. The mean ICC reached 0.88 (range, 0.753 to 0.999, *p* < 0.001) for the intraobserver and 0.86 (range, 0.752 to 1, *p* < 0.001) for the interobserver.

### Training, Validation, and Testing of the Radiomics Model

**Figure 4:**
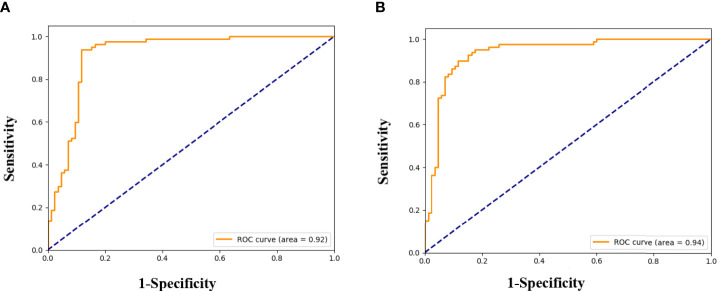
Receiver operating characteristic (ROC) curves for the prediction of ALNM in breast cancer in the validation **(A)**, and testing cohorts **(B)**.

We successfully developed the radiomics model to predict ALNM of breast cancer by integrating the radiomics features, which reflected the heterogenicity of different ALNs. All ROC curves are given in **Figure 4**. In our study, the average classification accuracy was 79.6%, with the best classification accuracy reaching 89.1% in the validation cohorts after 8,000 trainings and validations. The radiomics model with the highest classification accuracy, used for predicting ALNM of breast cancer, showed an AUC of 0.92 (95% CI: 0.91-0.93, *p*=0.002), a PPV of 95.9%, a NPV of 83.7%, a sensitivity of 82.4%, and a specificity of 96.3% in the validation data.

Testing results of the radiomics model also indicated its robustness. In the testing cohort, the model yielded a high AUC of 0.94 (95% CI: 0.93-0.95, *p*=0.005) and a classification accuracy of 88.5%, with a PPV of 89.3%, NPV of 87.7%, sensitivity of 88.2%, and specificity of 88.7% for identifying metastasis in an ALN. Details are given in **Table 4**.

**Table 4:** Performance of the radiomics model from the validation and testing cohorts for predicting ALNM of breast cancer.

	The validation cohorts	The testing cohorts
AUC	0.920 (95% CI 0.910-0.930)	0.940 (95% CI 0.930-0.950)
Accuracy	0.891 (95% CI 0.691-0.982)	0.885 (95% CI 0.712-0.960)
Sensitivity	0.824 (95% CI 0.671-0.890)	0.882 (95% CI 0.696-0.911)
Specificity	0.963 (95% CI 0.701-0.990)	0.887 (95% CI 0.699-0.948)
PPV	0.959 (95% CI 0.723-0.981)	0.893 (95% CI 0.746-0.977)
NPV	0.837 (95% CI 0.723-0.889)	0.877 (95% CI 0.726-0.902)

AUC, area under the curve; NPV, negative predictive value; PPV, positive predictive value; CI, confidence intervals.

## Discussion

ALNM is an important impact factor not only for prognosis but also for the treatment decisions for patients with breast cancer (25). To our knowledge, our study is the first attempt to construct a radiomics model by directly segmenting ALNs of patients, rather than tumor tissues, for predicting ALNM of breast cancer preoperatively. Furthermore, the large sample size was also a strength and the model demonstrated promising prediction performance. In our radiomics model for ALNM of breast cancer, the AUC was 0.92 (95% CI: 0.91-0.93, *p*=0.002) in the validation cohorts and 0.94 (95% CI: 0.93-0.95, *p*=0.005) in the testing cohorts with a PPV of 89.3%, NPV of 87.7%, sensitivity of 88.2%, and specificity of 88.7%. What this suggested is that our SVM model has great potential for predicting ALNM in breast cancer.

In recent years, many imaging techniques, such as US (7) and mammography (8), have played a significant role in detecting ALNM in patients with breast cancer. In addition, MRI and PET/CT (10, 11) have also been proposed for use in evaluating ALNM in breast cancer. In particular, breast MRI, which is widely used in the preoperative assessment of ALN status in breast cancer patients, exhibits better performance than some other techniques such as US and mammography (26). Compared with CT, MRI and PET/CT display improved sensitivity and specificity in the assessment of ALNM, with the sensitivities ranging from 47.1-98.5% and 48.1-85.7% and the specificities ranging from 57.8-98.0% and 61.5-94.7%, respectively (27–32). However, the diagnostic accuracy of MRI and PET/CT varies depending on the study. Although MRI offers the advantages of using no ionizing radiation and high quality soft tissue contrast, the long scan time and the effect of body position may increase patients’ discomfort. Besides, the greater number of contraindications associated with MRI and the higher cost of a PET/CT scan are also obstacles to their broad use compared with other methods. Given that CECT has a short scan time and high spatial resolution, patients readily accept this modality. And, more significantly, CECT not only displays the enhanced breast cancer tissue and ALNs, but can also exclude possible intrapulmonary and thoracic bone metastases. Besides, ALNs on CT images are clearly visible against the background of adipose tissue. Therefore, CECT has also been widely used for the preoperative evaluation of breast cancer (33–35). In general, lymph node size on the images serves as one of the main criteria for diagnosing malignant metastasis (36). However, a previous study reported that lymph node size was associated with ALNM in breast cancer but not found to be an independent predictor for ALNM (37). In fact, it is difficult to determine whether enlarged ALNs have metastasis only from the images, especially in the presence of multiple lymph nodes. Hence, clinically, there is a great need for a quantitative, non-invasive, and accurate method for preoperatively predicting ALNM in breast cancer. A recent study evaluated the predictive value of multidetector-row computed tomography for ALNM in breast cancer by integrating multiple CT signs, and achieved an AUC of 0.893 (38). In addition, some researchers found that real-time three-dimensional contrast-enhanced ultrasound (7) and mammography (8) could also be used for predicting ALNM in breast cancer, with diagnostic accuracies of 87.7% and 78.4%, respectively. However, these studies were based on subjective assessments and were greatly influenced by the operator’s experience such that the results may not be reproducible, and it may not be possible to achieve a precise location, qualitative evaluation, and quantitative analysis for all ALNs using this approach.

With the development of precision medicine and artificial intelligence, radiomics has appeared as a new medical field that is able to capture and quantify lesion heterogeneity, especially for lesions in oncology patients, by extracting and analyzing the quantitative texture features (39). Importantly, we have made great efforts and obtained some amazing results about radiomics analysis in recent years (40–42). In this study, we first extracted 396 radiomics features from each ALN in the CECT images, and used all of the radiomics features to develop the radiomics model for discriminating ALN status in patients with breast cancer based on the SVM algorithm. We obtained promising classification accuracies of 89.1% and 88.5% and AUCs of 0.92 (95% CI: 0.91-0.93, *p*=0.002) and 0.94 (95% CI: 0.93-0.95, *p*=0.005) in the validation and testing cohorts, respectively, outperforming evaluations made from traditional images, such as PET/CT (AUC = 0.847) (43) or MRI (AUC = 0.712) (28).

In recent years, many radiomics analyses have been explored for use in predicting LNM in breast cancer. In several previous studies based on MRI, the radiomics models achieved favorable predictive performance for ALNM in patients with breast cancer, with the AUCs reaching 0.863 (16), 0.869 (17), 0.819 (18), 0.861 (21), and 0.83 (22), but they are inferior to ours. In another study based on dynamic contrast-enhanced MRI (DCE-MRI), there was a favorable performance for the radiomics signature for predicting ALNM in breast cancer and distinguishing the number of ALNM (less than two positive nodes/more than two positive nodes), which achieved the highest AUC of 0.87, but was still inferior to our results (19). Moreover, a recently published study, which was based on CECT, used deep learning to predict SLNM in patients with breast cancer and also further distinguished the number of metastatic SLNs (the highest AUC was 0.817) (20). Of course, some radiomics models based on mammography (the highest AUC was 0.895) and US (the highest AUC was 0.84) have also been established as reliable and noninvasive tools for preoperative prediction of ALNM in breast cancer (23, 24), whose AUCs are inferior to ours too.

All in all, in these previous studies, all segmentations of the ROIs were focused on the tumor lesion itself for the radiomics analysis of ALNM in breast cancer. According to our knowledge, there has been no radiomics study published currently regarding the segmentation of ALNs themselves as ROIs for preoperatively predicting ALNM in breast cancer. Our study directly analyzed ALNs on the axial CECT images, and gave a higher AUC, which provided an outstanding advantage compared with previous methods. Radiomics analysis of the tumor itself could only estimate the status of ALNs in breast cancer, and it was unable to confirm accurately which lymph node had metastasis. In contrast, the radiomics analysis of ALNs as ROIs was much more helpful in locating the metastatic lymph nodes, which could guide an appropriate operation strategy and support personalized medicine.

Our study had several limitations. First, our study design was retrospective and the radiomics model for predicting ALNM was established on the basis of data obtained from a single center. Therefore, prospective multicenter studies including larger datasets are needed to further validate the robustness and reproducibility of our prediction model. Second, CT scans were adopted in our study, which employ ionizing radiation that may be harmful to patient health. However, the CT scan times were short, which can reduce the expense and discomfort of patients, and this method has fewer contraindications than MRI. Of course, an MRI-based or US-based radiomics model also needs to be developed to predict ALNM in breast cancer and compared with our CT-based model in the future. Third, our study only selected axial CECT images. It is necessary to further analyze axial non-enhanced CT scans or coronal/sagittal CECT images as well as the combination of the two or several scans, which would contribute to a better predictive performance of ALN status. Fourth, two-dimensional analysis, rather than three-dimensional analysis, was adopted in our study, which may lead to the loss of some information in the lesion. Therefore, we need to analyze the whole focus in future work. Fifth, the methods of data analysis may not be the most reasonable in our study. Hence, other optimization algorithms will be tried to develop better models for predicting ALNM of breast cancer in the future. Finally, more clinical, pathological, and radiological characteristics and even genomic information about the breast cancer patients should be taken into account in our radiomics model. Other models based on other modalities or clinical models will also be developed as a comparison in the future.

## Conclusion

In conclusion, the CECT-based radiomics model, which incorporated a radiomics signature, exhibited a powerful predictive performance and great potential for predicting ALNM in breast cancer. Thus, this model may facilitate clinical decision making and may improve survival outcomes in selected patients.

## Data Availability Statement

The original contributions presented in the study are included in the article/**Supplementary Material**. Further inquiries can be directed to the corresponding authors.

## Ethics Statement

This study complies with the guidelines for human studies. The research was conducted ethically in accordance with the World Medical Association Declaration of Helsinki. Ethical approval was obtained from the Affiliated Hospital of Southwest Medical University (KY2019182). According to the laws of our country, no patient contact or consent was needed. The collecting, analyzing, and restoring of the data were pseudonymized, and with high data security.

## Author Contributions

All authors contributed to the planning, writing and/or revising of this manuscript. All authors contributed to the article and approved the submitted version.

## Funding

This study was supported by the Technology Strategic Cooperation Project Between Luzhou Municipal People’s Government and Southwest Medical University (2019LZXNYDZ04), the Health Committee of Sichuan province (19PJ151), and the Project of Southwest Medical University (2020ZRQNA041).

## Conflict of Interest

The authors declare that the research was conducted in the absence of any commercial or financial relationships that could be construed as a potential conflict of interest.

## Publisher’s Note

All claims expressed in this article are solely those of the authors and do not necessarily represent those of their affiliated organizations, or those of the publisher, the editors and the reviewers. Any product that may be evaluated in this article, or claim that may be made by its manufacturer, is not guaranteed or endorsed by the publisher.

## Supplementary Material

The Supplementary Material for this article can be found online at: https://www.frontiersin.org/articles/10.3389/fonc.2021.726240/full#supplementary-material
