# Supplementary material for: Prediction of Metastasis in the Axillary Lymph Nodes of Patients With Breast Cancer: A Radiomics Method Based on Contrast-Enhanced Computed Tomography
Source: Front Oncol. 2021 Sep 20;11:726240. doi: 10.3389/fonc.2021.726240 (PMC8488257; doi:10.3389/fonc.2021.726240)
Supplement: Supplementary file 1 [file DataSheet_1.docx]

**The following is** **the details** **of inclusion and exclusion criteria for** **axillary lymph nodes (ALNs) in patients with breast cancer in our study.**

In order to match the pathological result of each ALN with its position in the CECT image accurately, the pathology and imaging of ALNs in all patients were discussed and confirmed by an experienced radiologist and an experienced pathological expert together. Only those ALNs with pathological results that exactly corresponded to their positions in the CECT images were recorded and included in our study. The following were some inclusion and exclusion criteria: (1) for patients in whom all ALNs (short diameter ≥ 0.5 cm) displayed metastasis, we included all of the ALNs for further texture extraction; (2) for some subjects whose ALNs (short diameter ≥ 0.5 cm) did not have metastasis, we also included all the ALNs of these subjects as the negative cohort; and (3) for other patients with metastasis in some of their ALNs (short diameter ≥ 0.5 cm), we first ranked the ALNs of each patient in a descending order according to the short diameter of the ALNs in the pathological sections and labeled them 1, 2, 3 etc. in turn. If the lymph nodes differed substantially in size and it was easy to match them with ALNs in the CECT images, all of those ALNs were included in the positive or negative cohort (e.g., if there were three ALNs with large differences in size and they could be identified easily in the CECT images, these three lymph nodes were included as part of the positive or negative group in our study). If one of the ALNs was obviously larger than the other lymph nodes and could easily be distinguished from the others among the images of multiple ALNs and there was little difference in the sizes of the other ALNs, this largest lymph node was included for further analysis but the remaining ALNs were excluded regardless of metastasis. If there was little difference in size among multiple ALNs (only some of them exhibited metastasis) and we distinguished them with difficulty, the patient was excluded. Besides, if the pathological results of the ALNs still failed to match their positions in the CECT images owing to other causes upon the discussion of the two experts (e.g., the short diameter of the lymph node on the pathological section was obviously different from that on the CECT image), which may lead to an inaccurate match, we excluded them.
